# Association of Stress-Related Factors With Anxiety Among Chinese Pregnant Participants in an Online Crisis Intervention During COVID-19 Epidemic

**DOI:** 10.3389/fpsyg.2021.633765

**Published:** 2021-04-30

**Authors:** Fangfang Shangguan, Ruoxi Wang, Xiao Quan, Chenhao Zhou, Chen Zhang, Wei Qian, Yongjie Zhou, Zhengkui Liu, Xiang Yang Zhang

**Affiliations:** ^1^School of Psychology, Capital Normal University, Bejing, China; ^2^School of Medicine and Health Management, Tongji Medical College, Huazhong University of Science and Technology, Wuhan, China; ^3^CAS Key Laboratory of Mental Health, Institute of Psychology, Chinese Academy of Sciences, Bejing, China; ^4^Department of Psychology, University of Chinese Academy of Sciences, Bejing, China; ^5^Shenzhen KangNing Hospital, Shenzhen, China

**Keywords:** prenatal anxiety, crisis intervention, perceived stress, pelvic pain, vaginal bleeding, COVID-19 outbreak

## Abstract

**Background:** Previous systematic review indicated the prevalence of prenatal anxiety as 14–54%. Pregnant women are a high-risk population for COVID-19. However, the prevalence of anxiety symptoms and related factors is unknown in Chinese pregnant women during COVID-19 outbreak.

**Objective:** To investigate the prevalence of anxiety symptoms and the related factors in Chinese pregnant women who were attending crisis intervention during the COVID-19 pandemic.

**Methods:** The data of this cross-sectional study were collected in about 2 months (February 28 to April 26, 2020). Data analysis was performed from April to May 2020. Participants completed a set of questionnaires via the Wechat Mini-program before starting the online self-help crisis intervention for COVID-19 epidemic. A total of 2,120 Chinese pregnant women who were attending a self-help crisis intervention participated in this study. A survey was developed to address possible stress-related factors in pregnant women during the COVID-19 outbreak, including demographic, socioeconomic, and pregnancy-related factors, as well as COVID-19 related factors. Generalized Anxiety Disorder-7 (GAD-7) scale and the 10-item perceived stress scale were, respectively, employed to measure anxiety and stress-related factors.

**Results:** A total of 21.7% (459) of pregnant women reported at least mild anxiety (≥5 on the GAD-7 scale), and only 82 women reported moderate to severe anxiety (≥10 on the GAD-7 scale). Factors associated with at least mild anxiety included living in Hubei province (*OR* = 1.68, *95% CI* = 1.32–2.13), nobody providing everyday life support (*OR* = 1.81, *95% CI* = 1.18–2.77), pelvic pain or vaginal bleeding (*OR* = 1.67, *95% CI* = 1.32–2.09), and higher perceived stress (*OR* = 6.87, *95% CI* = 5.42–9.02). Having relatives or neighbors with a diagnosis of COVID-19 was not associated with anxiety (*p* > 0.05).

**Conclusions and Relevance:** Our findings indicate that evaluation and intervention for maternal and infant health are necessary in pregnant women with anxiety during COVID-19 epidemic, especially those with higher perceived stress, less everyday life support, or vaginal bleeding. Interactions among these related medical, social and psychological factors need to be investigated in future studies.

## Introduction

Prenatal anxiety in pregnant women is a worldwide public health issue due to its high prevalence and heavy burden posed to not only pregnant women themselves but also their family. Previous studies have suggested that 14–54% pregnant women experienced anxiety (Madhavanprabhakaran et al., 2015; Rees et al., 2019; Adhikari et al., 2020; Bhushan et al., 2020; Molgora et al., 2020). A recent Chinese study found maternal anxiety in 26% of 119 well-educated and employed healthy pregnant women (Wu et al., 2020). Prenatal anxiety can result in adverse perinatal outcomes (Mirzadeh and Khedmat, 2020), impaired fetal brain development (Wu et al., 2020), and even long-lasting adverse health outcomes in their offspring's late lives (Helgertz and Bengtsson, 2019; Rees et al., 2019). During the present COVID-19 epidemic, a systematic review found that among 108 pregnant women between December 8, 2019 and April 1, 2020, 91% delivered by cesarean section (Zaigham and Andersson, 2020). The high rate of cesarean section may reflect the anxiety-related impacts on mothers under the COVID-19 outbreak estimated by researchers (Fakari and Simbar, 2020; Mirzadeh and Khedmat, 2020). However, the screening and recognition of anxiety symptoms during pregnancy remain insufficient (Bright et al., 2019; Hoyer et al., 2020).

Prenatal anxiety has been associated with socioeconomic factors, pregnancy-related factors, and perceived stress (e.g., Rallis et al., 2014; Kang et al., 2016). Several studies reported that anxiety during pregnancy was associated with low socioeconomic status (including income, education and employment status) (Kang et al., 2016; Adhikari et al., 2020; Liao et al., 2020). A previous study reported that pregnant women experienced fewer anxiety symptoms during the second trimester compared to the other trimesters (Rallis et al., 2014). Nulliparous women might be less anxious than multiparous women (Koelewijn et al., 2017; Liao et al., 2020; Lu et al., 2020). Furthermore, the lack of someone providing emotional support was associated with anxiety symptoms in pregnant women (González-Mesa et al., 2020). Besides, more anxiety in pregnant women was related to high perceived stress (Gul et al., 2017; Li et al., 2020). Perceived stress, the cognitive appraisal process when facing stressful situations, is closely related with prodromal stages of psychiatric disorders (Taylor, 2015).

COVID-19 epidemic may have exerted extra influence on prenatal anxiety in pregnant women (Corbett et al., 2020; Mirzadeh and Khedmat, 2020). First, pregnant women had heightened anxiety about health status of their family members during the epidemic than before (Corbett et al., 2020). Second, health education about COVID-19 have stressed chronic illness as high risk for complications in severe COVID-19 patients (Beghi et al., 2020; Bravi et al., 2020), therefore, pregnant women with a history of chronic illness may be more anxious than those without. Third, in China, participants in Hubei Province may be more anxious of being infected with COVID-2019 when compared with those in the non-endemic provinces (Yuan et al., 2020). However, there were only 33 participants from Hubei was included in that study, which were not convincing in explaining the anxiety level in Hubei population. Last but not least, during COVID-19 epidemic, pregnant women may also be anxious about the lack of accessibility of health service because of threatened miscarriage when experiencing vaginal bleeding (Hooker, 2020). Pregnant women were more preferentially admitted to a hospital during previous influenza epidemics, seeking high quality of medical care (Mertz et al., 2019). However, this year, pregnant women were anxious about possible COVID-19 risks in hospital settings, so some canceled regular visits in the hospital, or want selective cesarean section to terminate pregnancy (Fakari and Simbar, 2020; Gunnes et al., 2020; Ding et al., 2021). Therefore, pelvic pain or vaginal bleeding might be both pregnancy-related and COVID-19 related stressful events.

To our best knowledge, no prior study has measured anxiety in pregnancy during COVID-19 epidemic. Moreover, as a subjective appraisal of stress, perceived stress has not been integrated in previous studies that investigated socioeconomic or pregnancy-related characteristics in prenatal anxiety. More importantly, some factors might raise health risk in pregnant women during COVID-19 epidemic, such as history of chronic illness, COVID-19 diagnosis of family members or neighbors, and living in Hubei. Accordingly, the objectives of this study include: (1) to investigate the prevalence of anxiety symptoms, (2) to explore the demographic, pregnancy-related factors, COVID-19 related factors, and perceived stress that are associated with anxiety in pregnant women.

## Methods

### Procedure

All participants were recruited by the obstetric clinicians through the Wechat. The criteria for inclusion were: all participants were pregnant Chinese women, and they all registered in an online self-help intervention program targeting crisis intervention during the COVID-19 epidemic. Pregnant women completed a set of questionnaires on the Wechat Mini-program before the beginning of the online crisis intervention for COVID-19 epidemic. The 7-day self-help online intervention was designed according to some core strategies of Problem Management Plus (PM+), a low intensity psychological intervention (Dawson et al., 2015). The main purpose of the self-help intervention was introduced on the webpage, concentrating on stress reduction of the public. The intervention was arranged as 10–20 min per day in consecutive 7 days. Before they started the self-help intervention, participants saw themes of every day, namely Stability, Relaxation, Sense of control, Self-efficacy, Social support, Keeping healthy, and Hope, which might help them make decision whether they would like to complete the questionnaire and then start the intervention. The enrollment of participants was carried out according to the Declaration of Helsinki. All women provided informed consent. The Institutional Review Board of Institute of Psychology, Chinese Academy of Sciences, approved this study. The data were collected in about 2 months (February 28 to April 26, 2020) with the dissemination of the online intervention.

### Measures

#### The GAD-7 Scale

The GAD-7 scale is used to assess the severity of generalized anxiety disorder. Each item is scored as 0–3-point on a Likert scale (3 = “almost every day” and 0 = “not at all”). Scores on the GAD-7 scale ranges from 0 to 21. The Chinese version of GAD-7 scale has been widely used in China (He et al., 2010). The Cronbach's α coefficient of the GAD-7 scale was 0.86 in the current study. The participants were evaluated as with at least mild anxiety symptoms when the total scores ≥ 5 on the Generalized Anxiety Disorder-7 (GAD-7) scale (Spitzer et al., 2006). Therefore, this study divided all the subjects into two groups: anxiety group and non-anxiety group.

#### The 10-Item Perceived Stress Scale (PSS)

The PSS was used to evaluate one's level of perceived stress in terms of unpredictability, and overload (Cohen et al., 1983). Each item is scored on 0–4-point using a Likert scale (4 = very often and 0 = never). Six of the 10 items evaluate the frequency of negative thoughts, and the remained items evaluate the frequency of positive thoughts. The four positive items are reverse scored and the scores for all items are added up as a total score. The Chinese version of the scale demonstrates good reliability and validity (e.g. Ng, 2013). In the present study, the Cronbach's alpha value was 0.82 for the PSS.

#### Demographic Data and COVID-19-Related Factors in Pregnant Women

A survey was developed to address possible stressors in pregnant women during the COVID-19 outbreak. First, we collected sociodemographic data including age, gender, residential location during the outbreak, education, marital status, professional information, family annual income, and support for everyday life. Second, the survey also included questions about factors that might raise anxiety in pregnant women during COVID-19 epidemic: (1) pelvic pain or vaginal bleeding; (2) history of chronic illness, including diabetes, hypertension, hypothyroidism, hyperthyroidism pre- or during pregnancy; (3) contact history with COVID-19 indicated by infection in their family and neighbors; (4) living in Hubei.

#### Statistical Analysis

The normal distribution of each variable was examined by Shapiro-Wilk test, and none of the variables showed normal distribution (all *p* < 0.01). Descriptive statistics of the two groups (anxiety group and non-anxiety group) were calculated. Categorical variables (family annual income, marital status, etc.) were reported in percentages. Continuous variables (such as age, gestational age, education year, etc.) were expressed as median (Min, Max). The Mann–Whitney *U-*test or chi-square test was used to test the differences in these variables between the anxiety group and the non-anxiety group. Chi-square test was used to compare the prevalence of anxiety symptom among early, middle, and late pregnancy. A binary logistic regression analysis was performed to test the underlying factors associated with mild to severe anxiety (yes/no). Independent variables were variables that showed significant differences between anxiety group and the non-anxiety group in the previous mentioned Mann–Whitney *U-*test or chi-square test. SPSS Statistic 21.0 was applied to perform the analyses.

## Results

### Demographic Characteristics of Participants

Totally 2,139 pregnant women who are currently living in 15 cities in China participated in this study, and 2,120 of them submitted qualified questionnaires. It is worth noting that there were 693 participants in Hubei. The average age of the participants is 30.51 years (*SD* = 9.67). Among all the participants, 31.3% have an annual family income below 80,000 RMB, and 0.5% of households have an annual income over 1,000,000 RMB. Among all participants, 440 pregnant women were in the first trimester (≤12 weeks), and 1,203 were in the third trimester (≥25 weeks). All participants' privacy was guaranteed.

### Prevalence of Anxiety

Mild to severe anxiety was identified in 21.7% (459) of pregnant women in this study, who were categorized as anxiety group. In anxiety group, most of the women reported mild anxiety (17.8%, *n* = 377), and only 82 women reported moderate to severe anxiety. 22.7, 21, and 21.5% women in early (gestational age ≤ 12 weeks), middle (at 13–24 weeks) and late pregnancy (≥25 weeks) reported at least mild anxiety, and no significant differences were found in prevalence among early, middle and late pregnancy (χ^2^ = 0.44, *p* > 0.05). The prevalence of prenatal anxiety symptoms were 27.0 and 19.1% in and out of Hubei province, respectively.

### Comparison of Stress Correlates Between Women With and Without Anxiety

There were significant differences in age, education levels and the percentage of residence in Hubei province (Bonferroni corrected *ps* < 0.05) between non-anxiety group and anxiety group. More women in anxiety group had an annual family income <80,000 RMB than in non-anxiety group (Bonferroni corrected *p* < 0.05). Chronic illness during or prior to pregnancy and current oral medication on chronic illness was reported by more women in anxiety group than in non-anxiety group (Bonferroni corrected *ps* > 0.05). Pelvic pain or vaginal bleeding occurred more in anxiety group than in non-anxiety group (Bonferroni corrected *p* < 0.05). Having neighbors or relatives with a diagnosis of COVID-19 was not associated with anxiety (*p* > 0.05). See Table 1.

**Table 1:** Comparison of COVID-19-related factors between the participants with and without anxiety.

	**Non-anxiety** **(*n* = 1,661)**	**Anxiety** **(*n* = 459)**	***Z* or *χ^2^***	***p***
Age	30 (19, 45)	30 (20, 47)	−3.07^**^	0.002
Educated	16 (6, 23)	15 (6, 23)	−3.44^**^	0.001
Married	98.4% (1,634)	97.4% (447)	1.95	0.163
smoking	0.2% (4)	0.7% (3)	1.86	0.172
Drinking	1.7% (29)	2.4% (11)	0.82	0.365
Family annual income <80,000 Yuan	29.3% (487)	38.3% (176)	13.63^***^	0.000
Gestational week	26 (1, 42)	30 (1, 41)	−0.56	0.576
Nulliparae	54.6% (907)	55.1% (253)	0.04	0.845
Nobody providing support in everyday life	5.4% (89)	8.9% (41)	7.98^**^	0.005
Perceived stress	13 (1, 25)	19 (6, 37)	251.87^***^	0.000
Work as medical staff	6.1% (102)	6.8% (31)	0.23	0.632
Residence (Hubei)	30.5% (506)	40.7% (187)	17.24^***^	0.000
Relatives with a diagnosis of COVID-19	1.1% (18)	1.3% (6)	0.16	0.689
Neighbor with a diagnosis of COVID-19	1.1% (19)	0.9% (4)	0.25	0.618
Pelvic pain or vaginal bleeding	37.9% (629)	48.1% (459)	15.82^***^	0.000
History of chronic illness	16.8% (279)	20.9% (96)	4.19^*^	0.041
History of medical conditions related to pregnancy	11.2% (186)	13.3% (61)	1.53	0.216
Current medication for chronic illness	8.5% (142)	12.4% (57)	6.33^**^	0.012

*Categorized variables were presented as percentage (n). Continuous variables were presented as median (Min, Max)*.

*Z: outcome of Mann–Whittney U-test*;

* *p < 0.05*;

** *p < 0.01*;

*** *p < 0.001*.

### The Correlates of Anxiety in Pregnant Women

The results of binary logistic regression showed that elder age, living in Hubei, without anyone to turn to for support in everyday life, higher perceived stress, pelvic pain, or vaginal bleeding were significantly associated with at least mild anxiety (Table 2). Pregnant women living in Hubei were 1.68 times more likely to be anxious than those living in other provinces (*OR* = 1.68, *95% CI* = 1.32–2.13). Pregnant women were 1.81 times more likely to be anxious when there was nobody providing everyday life support (*OR* = 1.81, *95% CI* = 1.18–2.77). PSS scores ≥14 was regarded as indicating higher perceived stress (Monk et al., 2020). Pregnant women with higher perceived stress are 6.87 times more likely to be anxious than those with lower perceived stress (*OR* = 6.87, *95% CI* = 5.42–9.02). Pregnant women with pelvic pain or vaginal bleeding were 1.67 times more likely to be anxious than those without (*OR* = 1.67, *95% CI* = 1.32–2.09).

**Table 2:** Factors associated with prenatal anxiety.

**Variable**	***n* (%)**	**Wald *χ^2^***	**df**	**OR**	**95% CI**	***p***
**Age (years)**	30 (19, 47)	5.78	1	0.96^*^	0.94–0.99	0.016
**Education (years)**	15 (6, 23)	0.66	1	1.02	0.97–1.07	0.418
**Living in Hubei**		18.21	1	1.68^***^	1.32–2.13	0.000
Yes	693 (32.7)					
No	1,427 (67.3)					
**Family annual income < 80,000 Yuan**		2.77	1	1.24	0.96–1.61	0.096
Yes	663 (31.3)					
No	1,457 (68.7)					
**Nobody providing support in everyday life**		7.35	1	1.81^**^	1.18–2.77	0.007
Yes	130 (6.1)					
No	1,990 (93.9)					
**Perceived stress**		193.71	1	6.87^***^	5.24–9.02	0.000
Yes	1,074 (50.7)					
No	1,046 (49.3)					
**Current medication for chronic illness**		3.69	1	1.43	0.99–2.06	0.055
Yes	1,921 (90.6)					
No	199 (9.4)					
**Pelvic pain or vaginal bleeding**		19.07	1	1.67^***^	1.32–2.09	0.000
Yes	850 (40.1)					
No	1,270 (59.9)					
**History of chronic illness**		3.54	1	1.33	0.99–1.78	0.060
Yes	375 (17.7)					
No	1,745 (82.3)					

*Continuous variables are presented as Median (Min, Max)*.

* *p < 0.05*;

** *p < 0.01*;

*** *p < 0.001*.

## Discussion

To our best knowledge, the current study is the first to integrate socioeconomic factors, pregnancy-related factors, COVID-19-related stressful events, and perceived stress in a survey on prenatal anxiety. The main findings are as following: (1) the prevalence of prenatal anxiety symptom was 21.7%, and most of the anxious pregnant women reported mild anxiety; (2) higher perceived stress was a critical predictor of prenatal anxiety symptoms, not indicating specific stressful events; (3) anxiety symptoms were associated with pregnancy-related stressful events, including nobody providing emotional support and experiencing pelvic pain or vaginal bleeding, and the latter was a both pregnancy-related and COVID-19 related stressful event; (4) anxiety symptoms were associated with living in Hubei, but was not associated with the other COVID-19-related factors.

In the current study, we found that during the COVID-19 epidemic, the prevalence of anxiety symptom was 21.7% in Chinese pregnant women attending the crisis intervention, and the rate was 27.0% in the pregnant participants living in Hubei. Similarly, two survey in Wuhan, respectively reported that 20.8% (in February 2020; Ding et al., 2021) or 24.5% (in March 2020; Liu et al., 2020) pregnant women felt anxious during the COVID-19 epidemic. The reason of the various rates in these studies might be different self-rating scales, sampling periods. Other reasons might be differences in demographic and socioeconomic facets, since we found differences of age, education level, and family annual income between participants with and without prenatal anxiety, which is consistent with previous studies in China (Kang et al., 2016; Liao et al., 2020; Lu et al., 2020).

Due to the cross-sectional design, we could not conclude whether our participants had increased anxiety compared to the period right before COVID-19 pandemic. A study in Turkey found that among the 63 pregnant participants, women with mild anxiety decreased and women with moderate and severe anxiety increased after 2019-nCoV infection (Ayaz et al., 2020). Therefore, the public health emergency brought by the COVID-19 epidemic might have influenced the pregnant women all over the world (e.g., de Arriba-García et al., 2021; Saadati et al., 2021). An international prospective cohort study had just started to evaluate the impact of COVID-19 on pregnant women postpartum women over the next 6 month period in 14 countries (using GAD-7 when assessing anxiety symptoms) (Motrico et al., 2021).

Importantly, our results suggested living in Hubei was an independent factor that related to self-reported anxiety symptoms of pregnant women during the COVID-19 epidemic. This is reasonable since Hubei province was much more influenced by the crisis of COVID-19 epidemic than other provinces in China. Similarly, a survey on pregnant women in and showed that more women Wuhan (the capital of Hubei province) in Wuhan felt anxious compared to those in Chongqing (a big city in southwestern China) (24.5 vs. 10.4%) (Liu et al., 2020). Furthermore, we found that pregnant women with experience of pelvic pain or vaginal bleeding were more likely to report anxiety symptoms during COVID-19 epidemic. Vaginal bleeding or pelvic pain might be related to prenatal anxiety according to previous studies before the COVID-19 epidemic. For example, Richardson et al. (2017) claimed that the experience of vaginal bleeding and/or abdominal pain in early pregnancy was highly anxiogenic. Pelvic pain and vaginal bleeding may rise anxiety in pregnant women because their association with miscarriage (Kilfoyle et al., 2016) or diagnostic uncertainty (Richardson et al., 2017). Pelvic pain or vaginal bleeding was both pregnancy-related and COVID-19 related stressful events, as we mentioned above.

We also found prenatal anxiety was related to nobody providing support in everyday life. This result is similar with previous studies, which suggested that lack of emotional support was associated with anxiety in pregnant women. In China, most pregnant women were taken care of by their husbands or other family members, and this is also the case in our study. Therefore, it is reasonable to find that pregnant women were more likely to report anxiety when nobody could provide support for them in everyday life, particularly in case of quarantine during the COVID-19 epidemic. Besides, in the current study, no significant differences were found in prevalence among early, middle, and late pregnancy. However, a survey in Chinese pregnant women before the pandemic also reported a relatively high rate (20.6%) of anxiety in women at least 38 weeks into pregnancy, and the high rate were attributed to socioeconomic status and the third trimester (Kang et al., 2016). No existing survey on prenatal anxiety during COVID-19 epidemic reported the association of pregnancy trimester and prenatal anxiety.

Notably, we found that women with higher perceived stress were more likely to be anxious than those with lower perceived stress, which is in line with a recent study in women with recurrent pregnancy loss (Li et al., 2020). High perceived stress indicates people perceive their lives as excessively stressful relative to their capability to cope. Perceived stress showed significant relationship with physical and psychological symptoms in numerous studies (Hewitt et al., 1992; Beshai et al., 2016; Hjelm et al., 2017). The PSS examined women's general beliefs about stress without giving a list of specific events (Hewitt et al., 1992), so scores on the PSS in our study were not biased by the events related to pregnancy, COVID-19 epidemic and the recall of past life events.

## Limitations

There are several limitations in this study. First, this is a cross-sectional study, which is not sufficient to find risk factors or examine perceived change in anxiety during pregnancy. Second, there were only 82 pregnant women with moderate to severe anxiety in our survey, which restricted the application of our results in the explanation of severe prenatal anxiety during COVID-19 pandemic. Third, we found a small proportion of women who had relatives or neighbors with a diagnosis of COVID-19. This might be the reason that we found that COVID-19 diagnosis of relatives or neighbors was not associated with anxiety symptoms. Fourth, more questions should be designed to detect COVID-19-related stressful events. Fifth, self-report pelvic pain and vaginal bleeding may be not as reliable as the evaluation by doctors. Sixth, there were significant differences in age, education levels and family incomes between pregnant women with and without anxiety. Although other related factors were identified by adjusting these confounders in this study, future studies should better match these characteristics between groups. Seventh, family annual income varies in different provinces, so the subgroup of family annual income <80,000 Yuan was not suitable for every province. This restricted the application of our result on the criteria of family annual income. Eighth, the response rate was not available due to the internet technological problem of the newly developed Wechat Mini-program. Last, depending on dissemination of the crisis intervention program, pregnant women in this study were mainly from Hubei, Beijing and Gansu, and there were scarce participants in the other 12 provinces. Therefore, we cannot compare the prevalence of anxiety of our survey with that of other provinces and find more associate factors of anxiety in the COVID-19 pandemic.

## Conclusions

Anxiety in about one-fifth of pregnant women highlights the importance of instant distribution of clinical and mental health advice in the early stage of infectious disease epidemic. Both COVID-19-related and pregnancy-related factors were associated with anxiety in pregnant women seeking self-help online crisis intervention during COVID-19 epidemic. Furthermore, our study is the first to claim that general beliefs about stress might also be an independent factor associated with anxiety during COVID-19 epidemic. However, since there were only 3.9% pregnant women reported moderate to severe anxiety in our survey, we should be cautious when applying our conclusions in severe prenatal anxiety during the COVID-19 pandemic. Our findings have important clinical implications for medical and mental health service during and after the COVID-19 epidemic. First, measurement of perceived stress may be recommended in clinical obstetric practice and psychological crisis intervention. Second, integrated service should be considered in clinical obstetric setting during and after the COVID-19 pandemic. Since vaginal bleeding and/or abdominal pain may be anxiogenic, mental health service should also be provided for pregnant women seeking medical help for vaginal bleeding and/or abdominal pain in community hospitals, specialist hospitals or general hospitals. Third, pregnant women with less everyday life support should be supported by mental health in the future clinical practice, especially when facing public health emergencies. Last, systematic preventive interventions need to be exerted for anxiety during pregnancy, including socioeconomic measures, psychological and medical interventions.

## Data Availability Statement

The raw data supporting the conclusions of this article will be made available by the authors, without undue reservation.

## Ethics Statement

The studies involving human participants were reviewed and approved by The Institutional Review Board of Institute of Psychology, Chinese Academy of Sciences. The patients/participants provided their written informed consent to participate in this study.

## Author Contributions

FS: conceptualization, formal analysis, methodology, writing – original draft, writing – review, and editing. RW: conceptualization, writing – review, and editing. XQ: data curation, formal analysis, and writing – original draft. CZho and CZha: writing – original draft. WQ: data collection. YZ: methodology and data collection. ZL: conceptualization, methodology, project administration, and investigation. XZ: investigation, writing – review, and editing. All authors contributed to the article and approved the submitted version.

## Conflict of Interest

The authors declare that the research was conducted in the absence of any commercial or financial relationships that could be construed as a potential conflict of interest.
